# Clinical Characteristics of Malignant Melanoma in Southwest China: A Single-Center Series of 82 Consecutive Cases and a Meta-Analysis of 958 Reported Cases

**DOI:** 10.1371/journal.pone.0165591

**Published:** 2016-11-18

**Authors:** Jia Yu, Xue Luo, Hui Huang, Zhifang Zhai, Zhu Shen, Hui Lin

**Affiliations:** 1 Department of Dermatology, Southwest Hospital, Third Military Medical University, Chongqing, 400038, People's Republic of China; 2 Institute of Tropical Medicine, Third Military Medical University, Chongqing, 400038, People's Republic of China; University of Alabama at Birmingham, UNITED STATES

## Abstract

**Purpose:**

The present study determined the clinical characteristics and prognostic factors in patients with malignant melanoma based on a series of 82 cases from January 2009 to December 2014 in Southwest Hospital and a meta-analysis (including 12 articles) involving 958 patients in China.

**Materials and methods:**

The database elements included basic demographic data and prognosticators which were extracted from medical records. Statistical analyses of survival, and multivariate analyses of factors associated with survival were performed using the Kaplan—Meier method, and the Cox proportional hazard model, respectively. Literatures were identified through systematic searches in PubMed, Embase, the Cochrane Library, China National Knowledge Infrastructure (CNKI) and Weipu database (VIP) database for the period from inception to December 2015. The meta-analysis was conducted using R 3.1.1 meta-analysis software

**Results:**

In this series of 82 cases, the median age of the patients was 57.50 years. Melanoma was located in the foot in 79% of patients. Sixty-one patients (74.4%) were classified as stage II-III. Thirty-two patients (39.0%) had acral malignant melanoma, and 31 patients (37.8%) had nodular malignant melanoma. The clinical characteristics of melanoma were similar to those in areas outside southwest China (from results of the meta-analysis). The median survival time was 29.50 months. The 1-year, 3-year, and 5-year survival rates were 84.1%, 39.0% and 10.9%, respectively. COX regression following multi-factor analysis showed that ulcer, tumor boundary and lymph node metastasis were associated with prognosis.

**Conclusions:**

The clinical characteristics of melanoma in Chinese were different from those in Caucasians. Ulcer, tumor margins, and lymph node metastasis were significantly associated with prognosis. Immune therapy may prolong the median survival time of patients with acral melanoma, nodular melanoma, or stage I-III disease, although these differences were not statistically significant.

## Introduction

Malignant melanoma is derived from neural crest melanocytes and is frequently found in the skin, digestive tract, eyes, genitals and nasal cavity. The highest incidence of malignant melanoma is found in the skin. Early local and distant metastasis, and poor prognosis are clinical characteristics of malignant melanoma [1]. There are clear demographic and ethnic differences in malignant melanoma, such as incidence, etiology, and clinical characteristics [2]. Malignant melanoma is a common malignancy, and is frequently found in fair-skinned people in Western countries. The highest incidence of malignant melanoma is in Queensland, Australia [3]. As melanin is presented in the skin, the incidence of malignant melanoma is less frequent in Africa, Spain and Asia. However, if the populations in these areas developed malignant melanoma, their survival time would be significantly lower than that in Caucasians [4–6]. The incidence of melanoma in China was relatively low, accounting for 1%–3% among that of all malignant tumors. However, the number of new cases in China each year is more than 20,000 [7], and currently has the highest incidence in all malignant tumors, with an annual growth rate of approximately 3% -5% [8].

There is a huge difference in the pathogenesis and clinical characteristics of melanoma between Chinese and Caucasians. Firstly, the causes are different. Melanoma in Caucasian occurs in areas with excessive ultraviolet radiation. The etiology is associated with skin color and ultraviolet radiation intensity [9]. Melanoma in Chinese mainly occurs in the extremities. The cause remains unclear. Clinical experience has shown that improper processing (local stimulation by knife, salting, freezing, laser etc.) is an important factor inducing malignant nevi [8]. Secondly, the pathological types are different. Melanoma in Caucasians occurs in body skin, and the most common type is superficial spreading type [10]. Malignant melanoma in Chinese is mostly acromegaly and mucosal melanoma type [8]. Thirdly, the majority of Caucasian patients with malignant melanoma have early lesions, which are diagnosed at stage I [10]. The majority of Chinese patients with malignant melanoma are diagnosed at stage II or III [8]. Due to the low incidence of malignant melanoma in the Asian population and scarce large-scale clinical trials, the number of melanoma cases reported in Asia is limited. In order to better understand this highly aggressive and race-specific malignant tumor, more information on different races is needed.

Southwest Hospital is a first-class hospital in Southwest China. Due to the high level of medical care in this hospital many patients undergo diagnostic tests and treatment. Therefore, the cases of malignant melanoma selected from this hospital comprehensively reflect the incidence of malignant melanoma in Southwest China. In this study, we selected 82 patients with malignant melanoma treated at Southwest Hospital between 2009 and 2014. The epidemiological and clinical characteristics of these patients were summarized, and factors relevant to malignant melanoma prognosis were analyzed. In addition, a meta-analysis that included 12 articles involving 958 Chinese patients was conducted. The aim of this study was to understand the characteristics of malignant melanoma incidence and the related factors affecting patient prognosis in China.

## Materials and Methods

### Database Design

A database was prospectively designed for the current analysis after approval by the institutional review board of Southwest Hospital (Chongqing, China) prior to the retrieval and review of medical records. Database elements were based on published prognostic factors in addition to basic demographic data, and characteristics of the included patients (age at diagnosis, gender, and living habits, etc.), disease (anatomic location and stage), treatment (modality and type of surgery), and follow-up (time of disease recurrence/progression, patients' death, local control interval and disease-free survival).

### Ethics Statement

Our study was approved by the Ethics Committee of the Third Military Medical University (Chongqing, China). The protocol was approved by the institutional review boards of the participating institutions. All patient records/information was anonymized and de-identified prior to analysis.

### Patients and Staging Evaluation

Inclusion criteria: ncAll patients were first pathologically diagnosed with cutaneous malignant melanoma (CMM). ②CDiagnoses made reference to the latest (7th edition) American Joint Committee on Cancer (AJCC) Staging System for cutaneous melanoma. elPatients with cutaneous malignant melanoma were hospitalized in the Dermatology Department of Southwest Hospital from January 1^st^, 2009 to December 31^st^, 2014. At the same time, pathological specimens of each patient with melanoma were retained, and re-diagnosis was made by two doctors. ④ Clinical data of all the patients were complete.

Exclusion criteria: ① Patiens were only with clinical diagnosis liTumors in the skin transferred from other parts were removed, and non-pathological sections of the case were excluded were excludhad any other serious chronic illnesses or other visceral tumors thClinical data were not complete.

There were 7733 patients hospitalized in the Dermatology Department of Southwest Hospital from January 1^st^, 2009 to December 31^st^, 2014. The number of skin cancer cases were 622, while the number of patients with cutaneous malignant melanoma were 103. Finally, a total of 82 patients with cutaneous malignant melanoma were analyzed after excluding 11 re-visiting patients, 2 patients with melanoma metastasis from other parts, and 8 patients without pathological section.

### Data Analysis

Data were analyzed using Microsoft Excel 2007 (Microsoft Corporation, Redmond, WA, USA) and SPSS (SPSS 20.0; StatSoft Inc., Tulsa, OK, USA). Kaplan—Meier curves were constructed for survival analysis and the Log rank test was used to determine the differences in survival rate. After single factor analysis, statistically significant variables were used in the forward stepwise regression method and multivariable COX regression analysis. Significance was set at *p* < 0.05 for all analyses.

### Meta-analysis

#### Search Strategy

A systematic search for papers in English and Chinese was performed in five databases: PubMed (January 1946 to December 2015), Embase (January 1989 to December 2015), the Cochrane Library (January 1993 to December 2015), China National Knowledge Infrastructure (CNKI) (1999 to December 2015), and VIP (1989 to December 2015). We used the following keywords along with MeSH terms: malignant melanoma, cutaneous, China/Chinese. We conducted the literature search using computer-based retrieval, manual retrieval, and literature tracing. Inclusion and Exclusion Criteria. The PRISMA statement could be found in S1 Table.

The inclusion criteria for this study were as follows: ① published papers, ② subjects of the study were patients with cutaneous melanoma, ③ observational studies, ④ pathologic diagnosis was cutaneous melanoma, ⑤ the number of patients with cutaneous melanoma and their clinical characteristics were clearly reported. The exclusion criteria were as follows: ① reviews, meta-analyses and cohort studies that were not original, ② repeatedly published studies and data, ③ unclear case diagnosis, ④ the number of cases was less than 10.

#### Data extraction

Datawere extracted by two investigators(Jia Yu, Xue Luo) independently using a standardized data extraction form including years of publication, first author’s name, region,survey year, total sample size, age and survival rate. Decisions were disagreements about data were resolved by a third investigator(Hui lin)

#### Data Analysis

The meta-analysis was conducted using R 3.1.1 meta-analysis software. With significant heterogeneity among studies(P<0.1,I^2^>50%),the pooled prevalence and 95% confidence intervals (CIs) were calculated by the random effect model estimates, Otherwise, the fixed effect model would be used. For subgroup analysis according to age, gender, stage, histology and anatomic location.

## Results

### Case Reports

#### Clinical characteristics of patients with malignant melanoma

Our study included 82 patients with a male to female ratio of 1:1. Patients were aged 4–87 years with a median age of 57.50 years. In our study, time from skin lesions to diagnosis was the interval time between the diagnosis of a malignant skin tumor and the skin pigmentation, mass, ulcer and other pathological changes in the skin. The time was determined by asking the patients when they were received by the doctor, and recorded along with the medical records. We used medical record reading to extract this information for analysis. Median time from skin lesions to diagnosis was 24.0 months. The proportions of patients with clinical stage I, II, III and IV disease were 14.6% (*n* = 12), 35.4% (*n* = 29), 39.1% (*n* = 32), and 10.9% (*n* = 9), respectively. The distributions of the samples according to pathological types were divided into four groups: patients with lentigo maligna melanoma (10 cases, 12.2%), acral melanoma (32 cases, 39.0%), nodular melanoma (31 cases, 37.8%) and superficial spreading malignant melanoma (nine cases, 11.0%). 65 cases (79%) had malignant melanoma located in the foot, nine cases (11%) in the hand, four cases (5%) in the head and face, and four cases (5%) in the trunk(Table 1).

**Table 1:** General clinical characteristics of patients with malignant melanoma.

Characteristics	No.	%
Gender		
Male	41	50.0
Female	41	50.0
The median age at diagnosis (years) Median(Minimum- Maximum) 57.5 (4–87)		
Time of skin lesions to diagnosis(month)Median(Minimum- Maximum) 24.0 (1.0–422.0)		
TNM Staging Categories for Cutaneous Melanoma according to AJCC		
I	12	14.6
II	29	35.4
III	32	39.1
IV	9	10.9
Pathological histology		
Lentigo melanoma	10	12.2
Acral melanoma	32	39.0
Nodular melanoma	31	37.8
Superficial spreading melanoma	9	11.0
Anatomic location		
Foot	65	79.3
Hand	9	11.0
Head and face	4	4.9
Trunk	4	4.9
Ulceration		
With	31	37.8
Without	51	62.2
Clear tumor boundary		
With	74	90.2
Without	8	9.8
Tumor texture		
Hard	64	78.0
Soft	18	22.0
Satellite lesions		
With	16	19.5
Without	66	80.5
Abnormal secretions		
With	19	23.2
Without	63	76.8
Lymph node enlargement		
With	21	25.6
Without	61	74.4
Systemic or distant lymph node metastasis		
With	10	12.2
Without	72	87.8

Of the 82 patients with malignant melanoma, 31 (38%) had ulcers, 74 (90%) had clear boundaries, 64 (78%) had hard tumors, 30 (37%) had tenderness, 16 (20%) had satellite lesions, 19 (23%) had abnormal discharge, 21 (26%) had enlarged lymph nodes, and 10 (12%) had systemic or distant lymph node metastasis (Table 1).

#### The annual number and survival rate of patients with malignant melanoma

**Fig 1:**
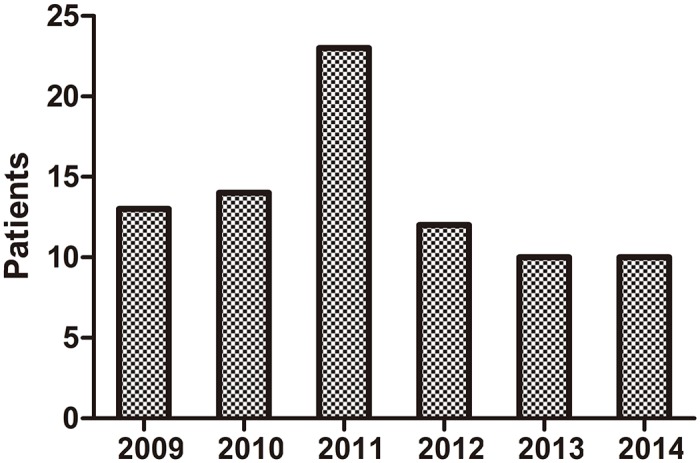
Trends in the number of melanoma patients hospitalized in the Dermatology Department in Southwest Hospital from January 2009 to December 2014.

**Fig 2:**
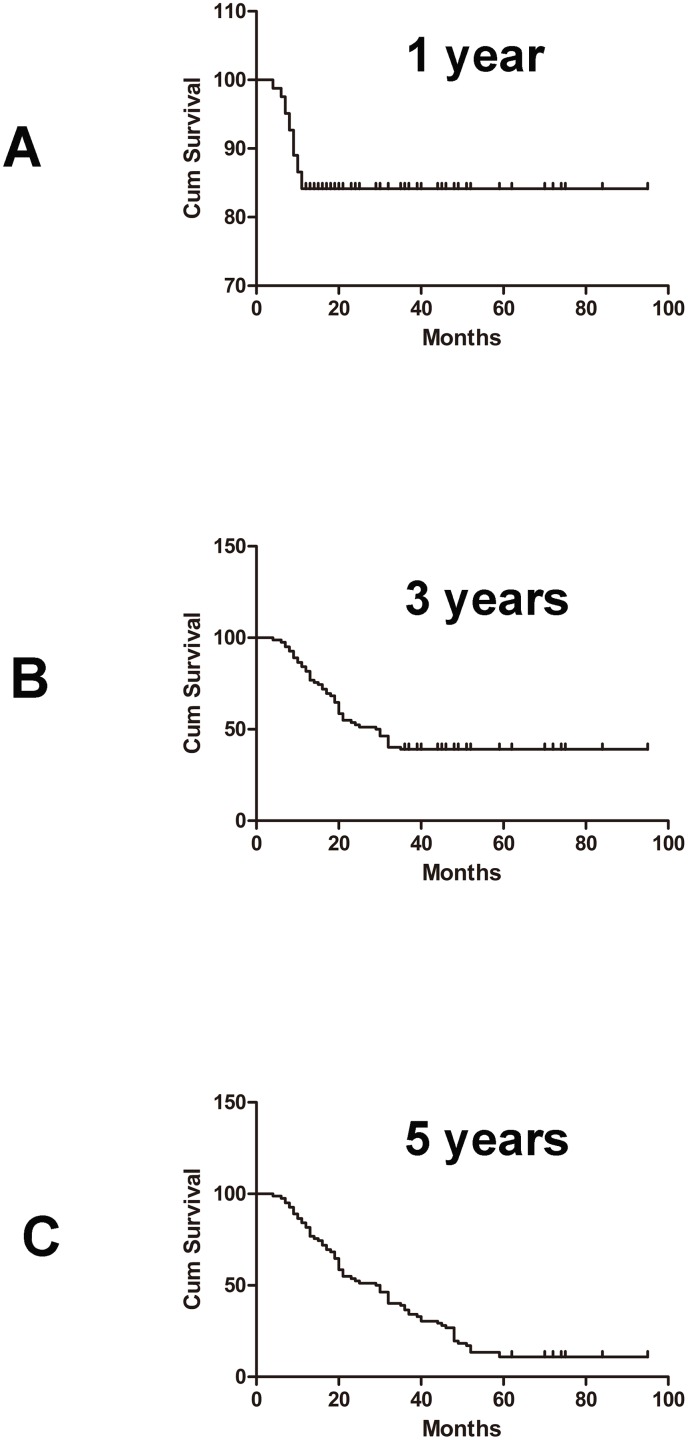
Kaplan—Meier analyses of overall survival for all patients according to the different stratums of prognostic factors (overall comparison was performed using the Mantel-Cox log-rank test). A. Overall survival at 1 year; B. Overall survival at 3 years; C. Overall survival at 5 years.

The number of melanoma patients hospitalized in the Dermatology Department in Southwest Hospital did not increase year by year (Fig 1). Median overall survival was 29.50 months (4–95 months). The 1-year, 3-year and 5-year survival rates were 84.1%, 39.0% and 10.9%, respectively (Fig 2).

#### Melanoma treatment

**Fig 3:**
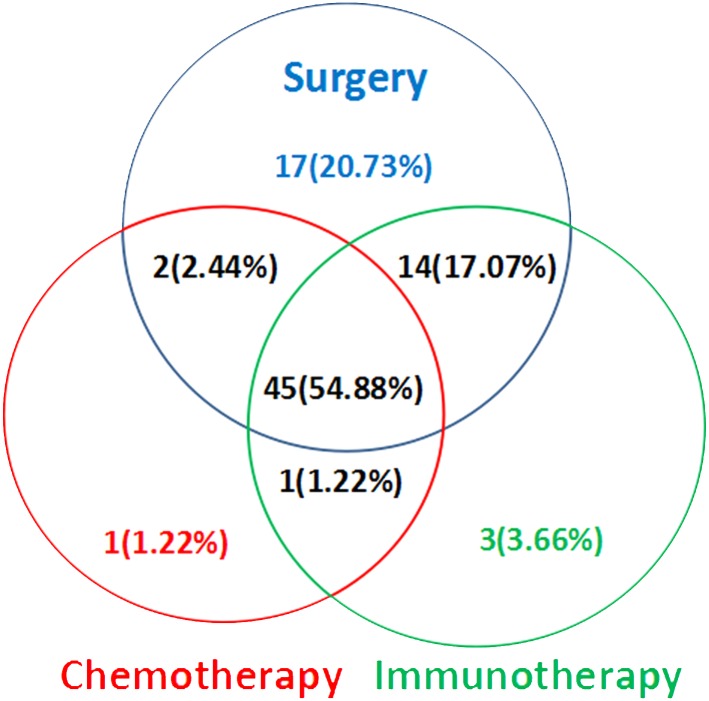
The melanoma treatments that were applied to these patients.

The selected cases received a variety of treatment modalities: surgery, chemotherapy and immunotherapy. All cases were treated with reference to the first edition of "*Consensus on the diagnosis and treatment of melanoma in China*" in August, 2008. On the basis of tumor resection and free flap transplantation, adjuvant chemotherapy and / or immunotherapy were selected according to the patient's condition. Chemotherapy drug was Dakaba, and immunotherapy drugs were the thymopentin and / or recombinant human interferon a-1 b. The specific dosage and course of chemotherapy and immunotherapy were selected according to the specific conditions of the patient. Among the 82 patients, 63 were treated with immunotherapy (including other treatment methods) (Fig 3).

#### Prognostic analysis

Univariate analysis showed that the pathological type, clinical stage (*p* = 0.001), ulcer (*p* = 0.004), tumor boundary (*p* = 0.023), tumor texture (*p* = 0.014), lymph node enlargement (*p* = 0.013) and systemic or distant lymph node metastasis (*p* = 0.001) were related to patient prognosis (Table 2). In our study, we intended to find the differences of survival rate and survival period of 1 year, 3 years and 5 years among different genders, age, stages and histology characteristics (Table 2). If there were any differences, it would suggest that the factor is the related factor with survival rate and survival period of 1 year, 3 years and 5 years. Mutual control was used in each group.

**Table 2:** Comparison of survival rate and median survival time among patients with different characteristics.

Characteristics	N	survival rate (n,%)						Survival time	
		1 year		3 years		5 years			
		n(%)	P	n(%)	P	n(%)	P	Median (Min- Max)	P
**Gender**									
Male	41	31(75.6)	0.034	13(31.7)	0.174	3(7.3)	0.482	20.0(4.0–84.0)	0.068
Female	41	38(92.7)		19(46.3)		6(14.6)		32.0(6.5–95)	
**Age (years)**									
≤57.50	41	34(82.9)	0.762	21(51.2)	0.041	5(12.2)	1.000	36.0(6.0–95.0)	0.164
>57.50	41	35(85.4)		11(26.8)		4(9.8)		21.0(4.0–84.0)	
**TNM Staging Categories for Cutaneous Melanoma according to AJCC**									
I	12	11(91.7)	0.007	9(75.0)	0.004	5(41.7)	0.003	50.0(11.0–74)	0.001
II	29	26(89.7)		13(44.8)		2(6.9)		32(7.0–95.0)	
III	32	28(87.5)		10(31.3)		2(6.3)		29.5(6.0–84.0)	
IV	9	4(44.4)		0(0.0)		0(0.0)		10.0(4.0–20.0)	
**Time of skin lesions to diagnosis (month)**									
-12	22	18(81.8)	0.935	8(36.4)	0.161	2(9.1)	0.430	25.0(4.0–28.0)	0.276
12–24	21	18(85.7)		5(23.8)		1(4.8)		21.0(7.0–27.0)	
24-	39	33(84.6)		19(48.7)		6(15.4)		35.0(7.0–95.0)	
**Pathological histology**									
Acral lentiginous melanoma	10	9(90.0)	0.459	4(40.0)	0.661	2(20.0)	0.543	33.5(8.0–75.0)	0.438
Mucosal melanoma	32	28(87.5)		12(37.5)		3(9.4)		24.0(6.0–84.0)	
Nodular melanoma	31	26(83.9)		14(45.2)		4(12.9)		32.0(4.0–950)	
Superficial spreading melanoma	9	6(66.7)		2(22.2)		0(0.0)		17.0(8.0–52.0)	
**Anatomic location**									
Foot	65	55(84.6)	0.220	26(40.0)	0.850	6(9.2)	0.122	30.0(4.0–95.0)	0.668
Hand	9	8(88.9)		4(44.4)		3(33.3)		35.0(11.0–74.0)	
Head and face	4	2(50.0)		1(25.0)		0(0.0)		14.5(7.0–51.0)	
Trunk	4	4(100.0)		1(25.0)		0(0.0)		27.5(17.0–52.0)	
**Ulceration status**									
With	31	22(70.9)	0.026	6(19.4)	0.005	2(6.5)	0.472	19.0(4.0–84.0)	0.004
Without	51	47(92.2)		26(50.9)		7(13.7)		36.0(6.0–95.0)	
**Clear tumor boundary**									
With	74	63(85.1)	0.606	27(36.5)	0.252	5(6.8)	0.004	27.0(4.0–95.0)	0.023
Without	8	6(75.0)		5(62.5)		4(50.0)		59.5(7.0–84.0)	
**Tumor texture**									
Hard	64	53(82.8)	0.723	21(32.8)	0.030	4(6.3)	0.021	22.5(4.0–95.0)	0.014
Soft	18	16(88.9)		11(61.1)		5(27.8)		44.0(9.0–84.0)	
**Satellite lesions**									
With	16	12(75.0)	0.270	5(31.3)	0.575	1(6.3)	0.681	19.0(4.0–84.0)	0.399
Without	66	57(86.4)		27(40.9)		8(12.1)		30.0(6.0–95.0)	
**Abnormal secretions**									
With	19	14(73.7)	0.167	3(15.8)	0.030	1(5.3)	0.667	19.0(7.0–70.0)	0.066
Without	63	55(87.3)		29(46.0)		8(12.7)		32.0(4.0–95.0)	
**Lymph node enlargement**									
With	21	17(80.9)	0.731	4(19.0)	0.038	1(4.8)	0.435	16.0(4.0–70.0)	0.013
Without	61	52(85.2)		28(45.9)		8(13.1)		32.0(6.0–95.0)	
**Systemic or distant lymph node metastasis**									
With	10	5(50.0)	0.007	0(0.0)	0.005	0(0.0)	0.592	11.5(4.0–20.0)	0.001
Without	72	64(88.9)		32(44.4)		9(12.5)		32.0(6.0–95.0)	
**Immunotherapy**									
With	63	55(87.3)	0.154	26(41.3)	0.448	6(9.5)	0.426	32.0(4.0–95.0)	0.390
Without	19	14(73.7)		6(31.6)		3(15.8)		14.0(7.0–75.0)	

Based on the univariate analysis, statistically significant survival factors related to malignant melanoma were assessed using Cox multivariate regression analysis. Tumor boundary (hazard ratio (HR) = 3.53; 95%confidence interval (CI) = 1.25–10.02), ulcer (HR = 2.09; 95%CI = 1.26–3.48) and lymph node metastasis (HR = 4.66; 95%CI = 2.14–10.14) were found to be related to patient prognosis (Table 3).

**Table 3:** Multivariate Cox regression analyses of survival factors in patients with malignant melanoma.

	B	SE	Wald	df	Sig.	Exp(B)	95.0% CI for Exp(B)	
							Lower	Upper
Tumor boundary	1.262	0.532	5.639	1	0.018	3.53	1.25	10.02
Ulceration status	0.738	0.260	8.057	1	0.005	2.09	1.26	3.48
Lymph node metastasis	1.538	0.397	15.013	1	0.000	4.66	2.14	10.14

#### Effect of immunotherapy on prognosis

The standard therapy for advanced melanoma is the use of a single drug (Dacarbazine); however, its efficiency is only about 10% [11]. As malignant melanoma is a highly immunogenic tumor, tumor immunotherapy may be a new approach for the treatment of malignant melanoma [12]. In our study, patients were divided into two groups: patients with immunotherapy and patients without immunotherapy. Patients with immunotherapy received a high dose of interferon alpha 2b (22 × 10^6^ U/day, 1–5 d / week) for 1 month. The median overall survival was 32 months (4–95 months) in 63 patients with immunotherapy, and was 14 months (7–75 months) in 19 patients without immunotherapy. Patients with immunotherapy had the longer median survival than patients without immunotherapy, but the difference between the two groups was not statistically significant (*p* = 0.390) (Table 2). We also analyzed the effect of immunotherapy as an independent factor on the overall survival rate with Cox regression analyses. Unfortunately, immunotherapy is not an independent factor in our study. Therefore, “immunotherapy” is not in Table 3. As malignant melanoma has high tumor immunogenicity, immunotherapy will likely be an important new treatment for malignant melanoma. However, immunotherapy is not an independent factor in our study. We think that the reasons may be the following two points. Firstly, sample size was smaller. Secondly, there are other confounding factors, such as treatments, different histology characteristics, and different stages. However, we have still compared clinical and pathologic findings between the patients treated with or without interferon alpha (Table 4). There were no statistically significant differences in the main clinical characteristics of the patients with and without immunotherapy.

**Table 4:** Clinical characteristics of the patients with and without immunotherapy.

	With immunotherapy (n = 63)	Without immunotherapy (n = 19)	*p*
Male (n, %)	31 (49.2)	10 (52.6)	0.794
Age (years) (median, min-max)	58.0 (31.0–87.0)	56.0 (4.0–81.0)	0.921
TNM Staging (AJCC) (n, %)			0.610
I	8 (12.7)	4 (21.1)	
II	24 (38.1)	5 (26.3)	
III	25 (39.7)	7 (36.8)	
IV	6 (9.5)	3 (15.8)	
Time from skin lesions to diagnosis (month) (median, min-max)	24.0 (1.0–422)	28.0 (6.0–156.0)	0.708
Pathological histology (n, %)			0.596
Lentigo melanoma	7 (11.1)	3 (15.8)	
Acral melanoma	27 (42.9)	5 (26.3)	
Nodular melanoma	22 (34.9)	9 (47.4)	
Superficial spreading melanoma	7 (11.1)	2 (10.5)	
Anatomic location			0.076
Foot	52 (82.5)	13 (68.4)	
Hand	4 (6.3)	5 (26.3)	
Head and face	3 (4.8)	1 (5.3)	
Trunk	4 (6.3)	0 (0.0)	

**Fig 4:**
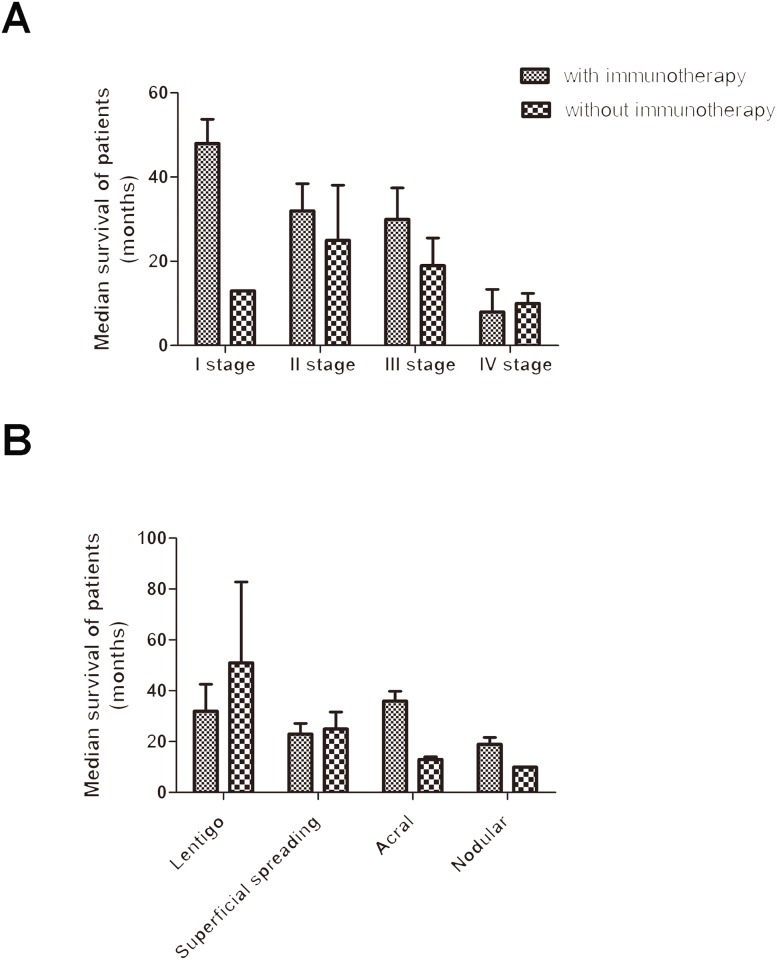
Effect of immunotherapy on median survival. A. Overall survival according to disease stage; B. Overall survival according to histology.

In patients with clinical stage I disease, the median survival was 48.0 ± 5.7 months in patients with immunotherapy, and was 13 months in patients without immunotherapy. In patients with clinical stage II disease, the median survival was 32.0±6.4 months in patients with immunotherapy, and was 25.0±13.1 months in patients without immunotherapy. In patients with clinical stage III disease, the median survival was 30.0±7.4 months in patients with immunotherapy, and was 19.0±6.5 months in patients without immunotherapy. In patients with clinical stage IV disease, the median survival was 8.0±5.3 months in patients with immunotherapy, and was 10.0±2.4 months in patients without immunotherapy (Fig 4A). Based on these data, immunotherapy can prolong the median survival of patients with stage I-III diseases, but the difference was not statistically significant.

In patients with lentigo maligna melanoma, the median survival was 32.0 ± 10.5 months in patients with immunotherapy, and was 51.0 ± 31.8 months in patients without immunotherapy. In patients with superficial spreading malignant melanoma, the median survival was 23.0 ± 4.1 months in patients with immunotherapy, and was 25.0 ± 6.6 months in patients without immunotherapy. In patients with acral melanoma, the median survival was 36.0 ± 3.8 months in patients with immunotherapy, and was 13.0 ± 1.0 months in patients without immunotherapy. In patients with nodular malignant melanoma, the median survival was 19.0 ± 2.6 months in patients with immunotherapy, and was 10 months in patients without immunotherapy (Fig 4B). In lentigo maligna melanoma patients, the median survival of patients without immunotherapy was longer than that of patients with immunotherapy. Immunotherapy had no significant effects on the median survival of patients with superficial spreading malignant melanoma. Immunotherapy prolonged the median survival of patients with acral melanoma and nodular melanoma. However, due to the small sample size, each group showed no significant differences.

### Meta-analysis

**Fig 5:**
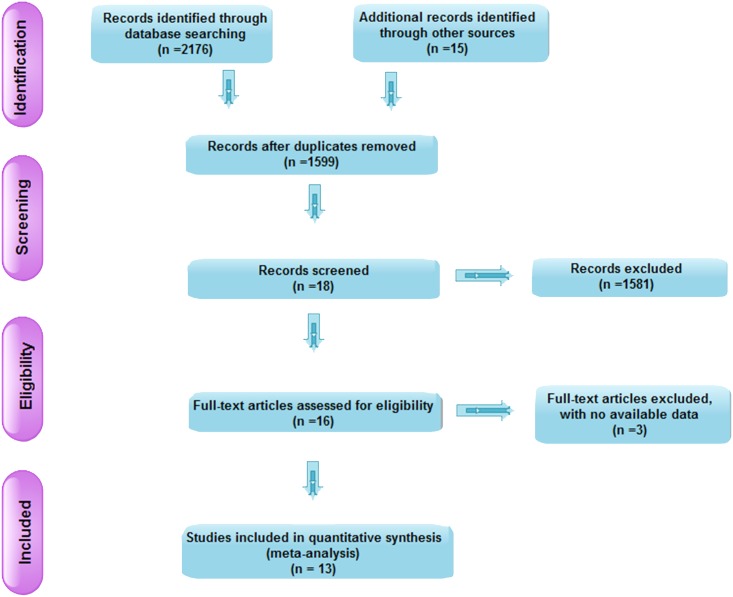
Flow chart of study inclusion.

A flow chart of the study selection process and exclusion criteria is shown in Fig 5. Twelve articles (involving 958 patients) were included in the meta-analysis. These patients were from Southwest China [13–24]. The characteristics of these articles are shown in Table 5.

**Table 5:** Main characteristics of all eligible studies.

Author (year)	Study period	Research region	Cases	Male:female	Age (year) Mean (min-max)
Teng (2003) (13)	1990.08–1998.04	Tianjin	85	53:32	49 (22–76)
Zhang (2008) (14)	1992.01–2002.01	Henan	45	26:19	52 (19–82)
Zhou (2008) (15)	1998.01–2007.01	Shanghai	29	17:12	55.2 (9–78)
Yang (2009) (16)	2000.10–2009.02	Shanxi	58	37:21	37 (26–78)
Lu (2010) (17)	1995.01–2003.01	Chengdu	62	38:24	52.2 (35–81)
Chi (2011) (18)	2006.01–2009.07	Beijing	357	195:162	51 (12–85)
Wang (2012) (19)	2009.07–2011.12	Zhejiang	23	15:8	57 (24–84)
Li (2012) (20)	1983.01–2010.01	Beijing	70	36:34	53.7 (12–83)
Du (2013) (21)	2010.01–2013.01	Xinjiang	33	17:16	62 (2–78)
Jiang (2014) (22)	2002.03–2012.03	Hebei	78	36:42	61.8 (45–70)
Xu (2014) (23)	2000.01–2014.01	Shenyang	78	35:43	56.5 (15–90)
Shi (2014) (24)	2009.1–2013.8	Henan	40	27:13	58.2 (35–80)

#### Gender

**Fig 6:**
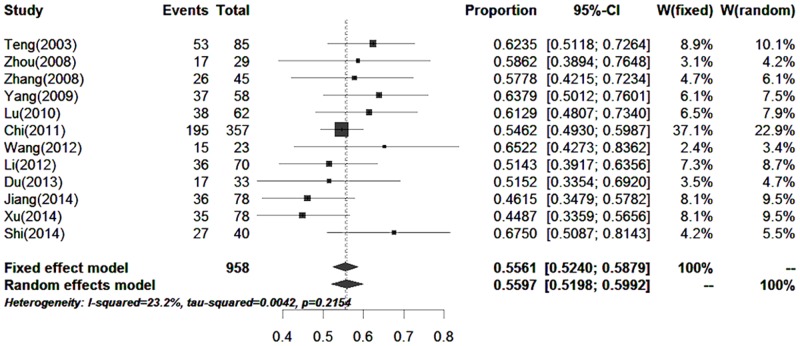
Forest plot of the proportions of males in the published data.

Twelve papers (including 958 cases) reported the proportion of skin melanoma in males and females. The proportion of males with skin melanoma was 55.61% (52.40%–58.79%) (Fig 6).

#### Stage

**Fig 7:**
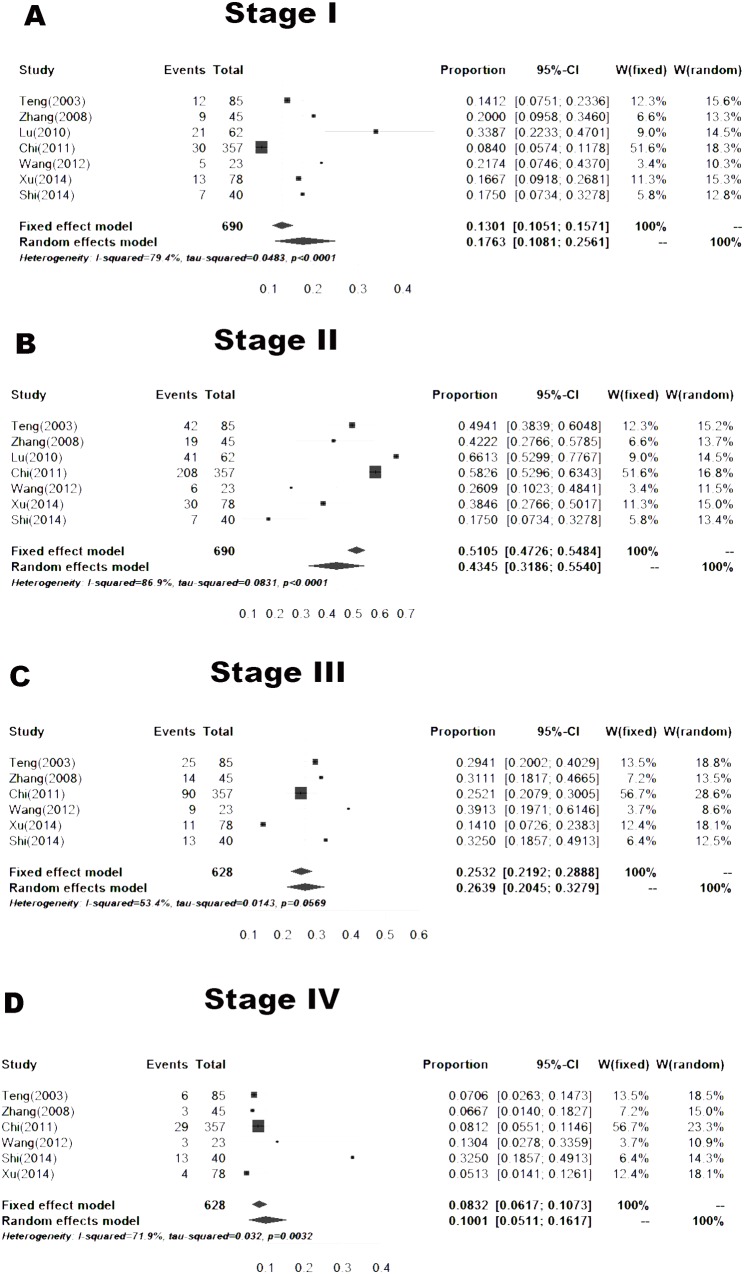
Forest plot of the proportions of different stages in the published data. A. Stage I, B. Stage II, C. Stage III, D. Stage IV.

Seven articles, including 690 cases, reported the proportions of skin melanoma at different stages in areas other than Southwest China. The proportions of skin melanoma at clinical stage I, II, III and IV disease were 17.63% (10.81–25.61%), 43.45% (31.86–55.40%), 26.39% (20.45–32.79%) and 10.01% (5.11–16.17%), respectively (Fig 7).

#### Histology

**Fig 8:**
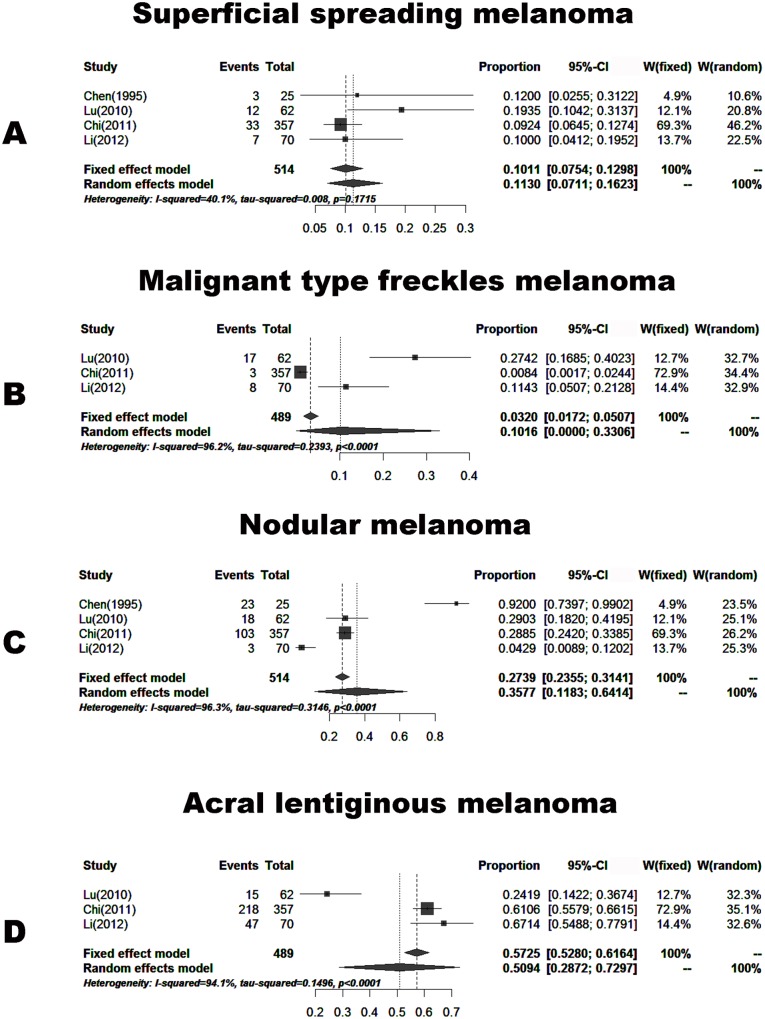
Forest plot of the proportions of histopathological diagnosis in the published data. A. Superficial spreading melanoma, B. Malignant type freckles melanoma, C. Nodular melanoma, D. Acral lentiginous melanoma.

Four papers (including 514 cases) reported the proportions of skin melanoma with different histology. These patients were from areas other than Southwest China. The proportions of skin melanoma according to pathological types were divided into four groups: patients with superficial spreading melanoma of 11.30% (7.11–16.23%), malignant type freckles melanoma of 3.2% (1.72–5.07%), nodular melanoma of 35.77% (11.83–64.14%) and acral lentiginous melanoma of 50.94% (28.72–72.97%) (Fig 8).

#### Anatomic location

**Fig 9:**
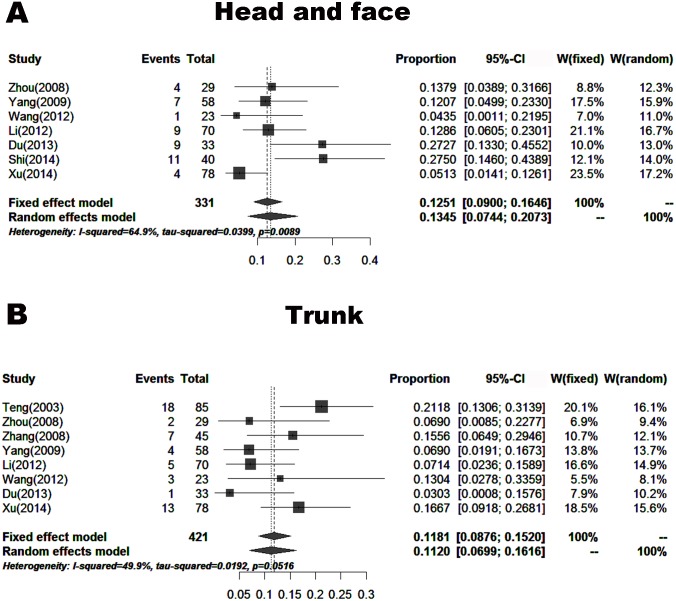
Forest plot of the proportions of melanoma located in the head and face, and trunk in the published data. A. Head and face, B. Trunk.

**Fig 10:**
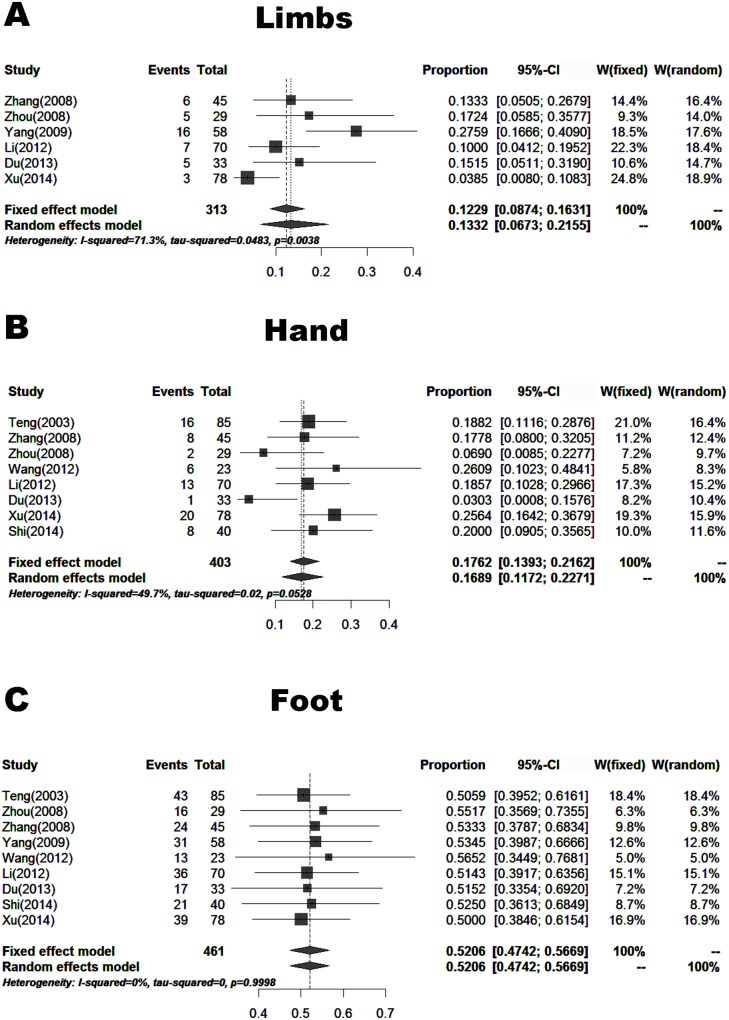
Forest plot of the proportions of melanoma located in the limb, hand and foot in the published data. A. Limb, B. Hand, C. Foot.

Nine papers (including 461 cases) reported the proportions of skin melanoma at different anatomic locations. These patients were from areas other than Southwest China. The proportions of skin melanoma were 13.45% (7.44–20.73%) located in the head and face, 11.20% (6.99–16.16%) in the trunk, 13.32% (6.73–21.55%) in the limb, 52.06% (47.42–56.69%) in the foot, and 16.89% (11.72–22.71%) in the hand (Figs 9 and 10).

#### Prognosis analyzed in other studies on Chinese melanoma

In our collected research articles on melanoma in China, there are only 6 articles involving the prognosis. These articles were summarized (Table 6). Because the quality of the literature is poor, this table is not adopted in our revised manuscript.

**Table 6:** The prognosis of eligible studies.

Author (year)	Research region	Cases	Male:female	Age (year) Mean (min-max)	Stage	survival rate
Teng (2003) (13)	Tianjin	85	53:32	49 (22–76)	I:12 II:42 III:25 IV:6	3 years I:91.7% II:58.9% III:16.7% IV:0.0%
Zhang (2008) (14)	Henan	45	26:19	52 (19–82)	-	120 months 44.4% (20/45)
Wang (2012) (19)	Zhejiang	23	15:8	57 (24–84)	I+ II:11 III:9 IV:3	32 months 87.5% (14/16)
Du (2013) (21)	Xinjiang	33	17:16	62 (2–78)	-	36 months 84.6% (11/13)
Jiang (2014) (22)	Hebei	78	36:42	61.8 (45–70)	I:3 II:48 III:27 IV:0	5 years 73.1% (57/78)
Xu (2014) (23)	Shenyang	78	35:43	56.5 (15–90)	I:13 II:30 III:11 IV:4	1 years I:88.8% II:69.6% III:20.0% IV:0.0%

## Discussion

The incidence of malignant melanoma in China is low compared with that in European and American countries. However, nearly 20,000 cases of cutaneous malignant melanoma are diagnosed in China each year [7]. Due to the low incidence of malignant melanoma in Asia and the lack of focus on melanoma, many Chinese patients with malignant melanoma have been characterized by distant metastasis and poor prognosis when they attend hospital [25]. Therefore, malignant melanoma is becoming an increasing public concern. To further understand the clinical characteristics and prognosis of malignant melanoma in China, it is necessary to clarify the epidemiological characteristics and risk factors affecting the prognosis of this disease.

Although the incidence of malignant melanoma in Southwest Hospital did not show an increasing trend year by year over the past 6 years, the clinical features of melanoma were different due to certain ethnic and regional factors. Survey data from studies on Caucasian patients with malignant melanoma showed that stage I patients accounted for 82%- 85%, and stage III patients accounted for 2%-5% [26–28]. Based on our study, the proportions of patients with clinical stage II and III disease were 35.4% (*n* = 29) and 39.1% (*n* = 32), respectively. These patients were from Southwest China. Based on the meta-analysis, the proportions of patients outside Southwest China with clinical stage II and III disease were 43.45% and 26.39%, respectively (Fig 6). Thus, the results were similar. The proportion of Chinese patients (including patients from Southwest China and outside Southwest China) with clinical stage IV disease exceeded 10%, which was much higher than the proportion of Caucasians (2%-5%). We speculated that the main reason for the higher proportion of Chinese patients with clinical stage IV disease was less understanding and emphasis on melanoma. It is hoped that Chinese patients with melanoma will attend hospital as early as possible. The proportions of patients with different pathological types were similar between patients from Southwest China (our study) and outside Southwest China (meta-analysis, Fig 7). The most frequent pathological types were nodular melanoma and acral lentiginous melanoma, which accounted for approximately 80%. However in Caucasians, the most frequent pathological type was superficial spreading melanoma [29]. Therefore, pathological types of melanoma differ among different ethnic groups. In our study, the male to female ratio was 1:1. However, the number of male cases was slightly bigger than that of females in the meta-analysis (Fig 5), which was similar in Caucasians. This difference may be attributed to our small sample size. There were obvious racial differences in the anatomic location of skin malignant melanoma. Exceeded 80% of the skin melanoma in Caucasians are located in the head and face. In our study, the most frequent anatomic locations were the foot and hand, which accounted for approximately 80%. The incidence of melanoma in Caucasians is related to skin color and ultraviolet radiation intensity. The pathogenesis of melanoma in Asians is still unclear. Based on our clinical experience, we suggest that inappropriate treatment (such as a knife cut, strangling, friction, etc.) can induce malignant transformation in pigment nevus.

Previous studies on the prognostic factors for malignant melanoma showed that gender, location, tumor size, depth of invasion, ulcer, staging and sentinel lymph node metastasis were related to patient prognosis. An agreement on whether gender, histological type and location are related to patient prognosis has not yet been reached [30–32]. In our study, the endpoint in 82 patients with malignant melanoma was overall survival. As this is a single-center retrospective study, sample size and available resources are limited. We only analyzed the correlations of age, gender, histological type, primary lesions, ulcer, primary tumor site, and treatment with prognosis. Prognosis by univariate analysis showed that pathological type, clinical stage, ulcer, tumor boundary, tumor texture, lymph node enlargement and systemic or distant lymph node metastasis were related to patient prognosis. Based on Cox multivariate regression analysis, tumor boundary, ulcer, and lymph node metastasis were related to patient prognosis. However, clinical stage and pathological type were not related to prognosis. But, Anna Plym [33] has reported that the prognosis of malignant melanoma was related to clinical stage and pathological type between young adults and older melanoma patients based on their cohort study. There was another report that acral melanoma patients have an inferior survival than patients with nonacral cutaneous melanoma in extremities when matched for stage [34]. There may be two reasons for the difference between our results and other reports. Firstly, the quantity of patients in this study is not enough. Secondly, there are many confounding factors in our study. Therefore, our study might cover up the truth. In the future, we will need to use a more advanced method to research.

Surgery was the major treatment choice for malignant melanoma. Whether surgery is the best choice to completely remove all lesions in melanoma patients with locally advanced or early disease and in patients with distant metastasis is unknown. Patients with distant metastasis may still be eligible for surgery, which is different from those with other malignancies [35–37]. Generally, melanoma is not sensitive to radiation; however, it is still an important treatment in special cases, such as for those with bone metastases, brain metastases, lymph node dissection and residual or recurrent head and neck melanoma (especially nasal melanoma) [38, 39]. Dacarbazine is a purine biosynthetic precursor, which can interfere with the biosynthesis of purine. When dacarbazine enters the body, liver microsomes interact with it by demethylation to induce cytotoxic effects. Dacarbazine is considered as the "gold standard" in melanoma chemotherapy. Treatments with new drug for advanced melanoma are needed in order to perform controlled studies [11]. For patients with advanced melanoma, chemotherapy is generally ineffective. Therefore, new treatment strategies are particularly important. As malignant melanoma has high tumor immunogenicity, biological therapy will likely be an important new treatment for malignant melanoma. In patients with stage I-III disease, immunotherapy had a definite effect according to evidence-based medicine; however, immunotherapy in patients with stage IV disease remains controversial [39, 40]. Cui reported on twenty-one patients with melanoma liver metastases treated with immunotherapy. Seventeen of these twenty-one patients were evaluated. One achieved complete remission, one achieved partial remission, six had stable disease and nine had progressive disease. The disease control rate was 47%, with a median progression-free survival of 3.7 months and a medium overall survival of 6 months [41]. In our study, immunotherapy prolonged patient survival. In patients who received immunotherapy, median survival was 32.0 months (4.0–95.0 months). In patients who did not receive immunotherapy, median survival time was 14.0 months (7.0–75.0 months). However, the difference was not statistically significant (*p* = 0.390). Immunotherapy prolonged the median survival of patients with stage I-III disease, but the difference was not statistically significant. In patients with lentigo maligna melanoma, median survival in patients without immunotherapy was longer than that in patients with immunotherapy. Immunotherapy had no significant effects on the median survival of patients with superficial spreading malignant melanoma. Immunotherapy prolonged the median survival of patients with acral melanoma and nodular melanoma. However, due to the small sample size, each group showed no significant difference. Therefore, a large sample is needed for subsequent validation. Pigmentation plays an important role in the development of melanoma. Melanins possess radioprotective and scavenging properties, and its presence can affect the behavior of melanoma cells. The presence of melanin in metastatic melanoma cells decreases the outcome of radiotherapy, and melanin synthesis is related to higher disease advancement. Based on previous cell-based and clinical research, it could suggest that inhibition of melanogenesis can improve radiotherapy modalities [42, 43]. The active melanogenesis could not only impair the cytotoxic action of cyclophosphamid but also has potent immunosuppressive properties. Thus, the inhibition of melanogenesis might represent a valid therapeutic target for the management of advanced melanotic melanomas [44]. Based on previous clinical research, melanogenesis shortens overall survival and disease-free survival in patients with metastatic melanoma. Inhibition of melanogenesis appears a rational adjuvant approach to the therapy of metastatic melanoma [45]. There might be other factors (melanogenesis) that influence the effects of immune therapy. The active process of melanogenesis generates reactive oxygen species as well as quinone and semiquinone intermediates that display cytotoxic, genotoxic, or mutagenic activities and act as potent immunosuppressants. The immunosuppression is best illustrated by ex vivo by shutting off T- and B-cell immune activities or the selective lymphotoxic effects of levodopa or products of its autoxidation. In melanotic melanoma cells, the process of melanin synthesis is deregulated, affecting the behavior of not only melanoma cells but also its surrounding environment. The net effect is cytotoxicity to surrounding tissues (but not to melanoma cells), mutagenesis in melanoma cells, and almost complete local immunosuppression. This inhibits the host responses and promotes tumor progression. In support of the latter, recent studies have shown that melanogenesis shortens overall and disease-free survival in patients with metastatic melanoma. The immunosuppressive field generated by intermediates of melanogenesis will attenuate any type of immunotherapy against melanoma. Note that intermediates of anogenesis can enter the circulation and have systemic effects, depending on the activity of the pathway and the tumoral volume. A clinical example of the latter is the general melanosis that can develop in some patients with metastatic melanotic melanoma [46].

Although immunotherapy has good therapeutic effect on the treatment of melanoma, immune-based therapy has its limitations owing to the tumor’s ability to generate an immunosuppressive environment [46]. Therefore, inhibition of melanogenesis appears to be a rational adjuvant approach to the therapy of metastatic melanoma [47].

## Conclusions

From January 2009 to December 2014, the number of melanoma patients hospitalized in the Dermatology Department in Southwest Hospital did not increase year by year. However, the clinical features of melanoma differed in ethnicity and region. Ulcer, tumor margins, and lymph node metastasis were significantly associated with the survival of melanoma patients. Tumor staging and classification were not correlated with survival in melanoma patients. Immunotherapy prolonged median survival in patients with acral melanoma, nodular melanoma and stage I-III disease. However, due to the small sample size of our study, these differences were not statistically significant. As our data were obtained from a single center with a small sample size and based the lower quality of the literature, these conclusions require to be confirmed by further studies.

## Supporting Information

S1 Table PRISMA statement. (DOC)
